# Dying cell clearance and its impact on the outcome of tumor radiotherapy

**DOI:** 10.3389/fonc.2012.00116

**Published:** 2012-09-11

**Authors:** Kirsten Lauber, Anne Ernst, Michael Orth, Martin Herrmann, Claus Belka

**Affiliations:** ^1^Department of Radiotherapy and Radiation Oncology, Ludwig Maximilian University of MunichMunich, Germany; ^2^Department of Internal Medicine 3, Friedrich-Alexander-Universität Erlangen-NürnbergErlangen, Germany

**Keywords:** Radiotherapy, apoptosis, necrosis, necroptosis, senescence, mitotic catastrophe, dying cell clearance

## Abstract

The induction of tumor cell death is one of the major goals of radiotherapy and has been considered to be the central determinant of its therapeutic outcome for a long time. However, accumulating evidence suggests that the success of radiotherapy does not only derive from direct cytotoxic effects on the tumor cells alone, but instead might also depend – at least in part – on innate as well as adaptive immune responses, which can particularly target tumor cells that survive local irradiation. The clearance of dying tumor cells by phagocytic cells of the innate immune system represents a crucial step in this scenario. Dendritic cells and macrophages, which engulf, process and present dying tumor cell material to adaptive immune cells, can trigger, skew, or inhibit adaptive immune responses, respectively. In this review we summarize the current knowledge of different forms of cell death induced by ionizing radiation, the multi-step process of dying cell clearance, and its immunological consequences with special regard toward the potential exploitation of these mechanisms for the improvement of tumor radiotherapy.

Radiotherapy is an essential treatment option for various types of cancer due to its profound potential to kill malignant cells and to abrogate clonogenic survival. Ionizing radiation, including X-rays, gamma rays, and heavy ions, induces damages in the cellular DNA, which lead to the activation of a highly sophisticated and finely tuned signaling cascade designated the DNA damage response (DDR) and – depending on the extent of damage – to transient or permanent cell cycle arrest, and/or cell death, respectively.

## THE DNA DAMAGE RESPONSE

The DDR is orchestrated by two conserved protein kinases, ATM (ataxia telangiectasia, mutated) and ATR (ATM and Rad3 related), which in concert control the cellular response to DNA double strand breaks (DSBs) and single-stranded (ss) DNA (Smith et al., 2010). ATM is recruited to DSBs by the Mre11–Rad50–Nbs1 (MRN) complex and phosphorylates the histone H2 variant H2AX, thus generating an interaction platform for other DDR constituents required for DSB repair (Shiloh, 2006). Concomitantly, ATM activates the DNA damage checkpoint by initiating the resection of the broken strand(s) leading to the generation of ssDNA, a DNA damage repair intermediate which, in turn, activates ATR kinase (Hurley and Bunz, 2007). ATR and ATM phosphorylate and activate two respective effector kinases termed Chk1 and Chk2. Collectively, these four protein kinases instigate multiple cellular pathways culminating in transient or permanent cell cycle arrest, DNA damage repair, and/or cell death (Jackson and Bartek, 2009). A crucial target of the ATM/ATR cascade is the tumor suppressor protein p53, a transcription factor, whose function is lost or compromised in more than 50% of all cancers (Levine, 1997). Under steady-state conditions, p53 protein is sustained at very low levels, since it is continuously being ubiquitinylated by the Mdm2 ubiquitin ligase and thus targeted for subsequent proteolytic degradation via the 26S proteasome. Yet, phosphorylation of p53 by kinases of the ATM/ATR pathway induces the dissociation of p53 from MDM2, and the subsequent reduction in p53 ubiquitinylation leads to a deceleration of proteasome-mediated degradation and hence a stabilization of p53 (Meek, 2009). Depending on the cell cycle phase and the type as well as the extent of DNA damage, p53 can function as a modulator of the DNA damage repair process, or as a transcriptional activator of genes, which are involved in transient or permanent cell cycle arrest, and/or cell death, respectively (Sengupta and Harris, 2005).

## APOPTOSIS

**FIGURE 1:**
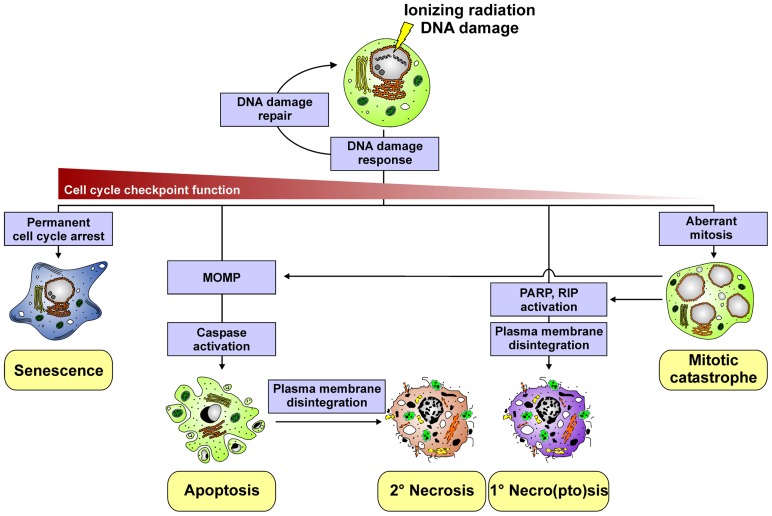
**Different cell death modalities induced by ionizing radiation**.

Apoptosis is one type of programed cell death. It is commonly considered to be the prevalent form of cell death underlying daily tissue regeneration and renewal. Morphologically, it is characterized by cellular shrinkage, chromatin condensation, nuclear fragmentation, and membrane blebbing (**Figure 1**). In response to radiotherapy, apoptosis is predominantly observed in cells of the hematopoietic system, and it is critically regulated by the mitochondrial, intrinsic death pathway (Rudner et al., 2001; Eriksson and Stigbrand, 2010). The pivotal events in this context involve the permeabilization of the mitochondrial outer membrane (MOMP) and the release of various proteins, including cytochrome *c*, from the mitochondrial intermembrane space into the cytosol, thus stimulating the formation of the apoptosome and the activation of procaspase-9. Activated caspase-9, in turn, triggers the activation of downstream effector caspases, which execute the final stages of apoptosis and the disintegration of the cell (Taylor et al., 2008). Crucial regulators of mitochondrial permeabilization and cytochrome *c* release are proteins of the B cell lymphoma-2 (Bcl-2) family, including the pro-apoptotic BH3-only (e.g., Puma) and the anti-apoptotic (e.g., Bcl-2) family members, which control MOMP via their impact on the oligomerization of the effector members Bax and Bak (Youle and Strasser, 2008). p53 links this signaling pathway to radiation-induced DNA damage by transactivating the expression of pro-apoptotic Bcl-2 family members, such as Puma and Noxa (Sengupta and Harris, 2005). Apart from the intrinsic pathway, apoptosis can be induced extrinsically via the ligation of death receptors, such as CD95 or the TRAIL receptors 1 and 2, by their corresponding ligands (Debatin and Krammer, 2004). Receptor clustering leads to recruitment and activation of the pro-caspases-8 and -10, triggering of the caspase cascade, and thus to apoptosis. Various proteins of the death receptor pathway are known to be upregulated in response to ionizing radiation (p53-dependently as well as -independently) and thus might contribute to apoptosis induction (Belka et al., 1998; Haupt et al., 2003). However, the intrinsic death pathway appears to be the major signaling mechanism of irradiation-induced apoptosis (Rudner et al., 2001). Notably, although p53 essentially controls the expression of various key regulators of apoptosis, irradiation-induced apoptosis can be observed in cancer cells with defective p53 function. Here, mechanisms, such as p63-/p73-dependent induction of pro-apoptotic Bcl-2 members and p53-independent stimulation of death receptor signaling have been described to be involved (Afshar et al., 2006; Wakatsuki et al., 2008).

## NECROPTOSIS AND NECROSIS

In tumor cells of epithelial origin, which reveal limited apoptosis induction in response to radiotherapy, radiation-induced DNA damage – especially when combined with hyperthermia - has been reported to stimulate necroptosis (Mantel et al., 2010; Schildkopf et al., 2010; **Figure 1**). The crucial events in this context include the hyperactivation of the DNA repair enzyme poly-ADP-ribose-polymerase (PARP) and the subsequent and substantial depletion of intracellular ATP levels (Vandenabeele et al., 2010; Vanlangenakker et al., 2012). This – in a so far poorly understood way – couples to the activation of receptor interacting protein (RIP), the formation of the high-molecular weight necrosome, and finally the execution of necroptosis as characterized by the production of reactive oxygen species (ROS), lipid peroxidation, swelling of organelles, rupture of the plasma membrane, and release of intracellular contents (Vandenabeele et al., 2010). Apart from necroptosis, ionizing radiation – particularly when applied in high single doses during ablative radiotherapy – can trigger necrosis, an accidental, uncontrolled form of cell death as a consequence of excessive physico-chemical stress (Vandenabeele et al., 2010). Moreover, secondary necrosis can occur when apoptotically dying cells are not properly and timely engulfed by neighboring cells or professional phagocytes, respectively (Munoz et al., 2010a; Silva, 2010). This is of specific relevance when the local phagocytic compartment is overwhelmed due to massive apoptosis induction in the context of tumor radiotherapy. In both cases the integrity of the plasma membrane is lost and cellular contents, often in an oxidatively modified and partially degraded form, leak into the surrounding tissue.

### MITOTIC CATASTROPHE

Mitotic catastrophe is a form of cell stress, which occurs in the context or as a result of aberrant mitosis owing to uncoordinated or improper entry into mitosis. It has been assigned to be the major death mechanism in response to irradiation-induced DNA damage of cells with defects in cell cycle checkpoints and impaired DNA repair mechanisms (e.g., cells with defective p53). In the course of mitotic catastrophe the formation of giant cells can be observed with aberrant nuclear morphology, centrosome hyperamplification, and multiple nuclei, and/or several micronuclei (**Figure 1**). These cells may survive for days, transit into senescence, or die by delayed apoptosis or delayed necro(pto)sis, respectively (Eriksson and Stigbrand, 2010).

## SENESCENCE

Radiation-induced senescence is a condition of permanent cell cycle arrest, which can be observed in cells, where DNA damage is excessive and cell cycle checkpoints are still intact (**Figure 1**). The hallmarks of cellular senescence include an enlarged and flattened cellular morphology, increased granularity, upregulation of cyclin-dependent kinase inhibitors, such as p16^INK4a^, p21^Waf1^, and p27^Kip1^, and positive staining for the senescence-associated β-galactosidase (SA-β-Gal). The key players in this scenario are p53 and pRB, yet senescent phenotypes have also been reported in the absence of functional p53 (Nardella et al., 2011). Senescent cells exit the cell cycle and do not further undergo cell division, but may remain metabolically active. Interestingly, they have been shown to release factors, which can be tumor suppressing as well as tumor promoting, and which can alter the immune response (Kuilman and Peeper, 2009; Coppe et al., 2010).

## DYING CELL CLEARANCE

Higher organisms have developed impressively efficient mechanisms of dying cell clearance as can be appreciated from the fact that dying cells are rarely to be observed in normal tissues, although – according to careful estimations – approximately one million cells per second undergo apoptosis in the course of everyday tissue turnover and regeneration (Reed, 2006). These cells are swiftly phagocytosed and degraded, and the immune system had to acquire the capability to distinguish them from cells, which are dying in the context of an infection. Whereas several types of amateur phagocytes, including fibroblasts, endothelial cells, and mesothelial cells, have been described, professional phagocytes, such as macrophages and dendritic cells (DCs), apparently play a crucial role in this scenario, particularly when the local amateur phagocytic compartment is overwhelmed (Lauber et al., 2004; Ravichandran, 2011; Wagner et al., 2011). Macrophages and DCs serve as professional dying cell scavengers with discrepant tasks. While tissue resident macrophages can proficiently engulf and degrade huge amounts of dying prey cells, DCs act as sentinels, which are highly potent in presenting and cross-presenting antigens, thus stimulating, skewing, or inhibiting adaptive immune responses, respectively (Steinman, 2007; Biswas and Mantovani, 2010).

**FIGURE 2:**
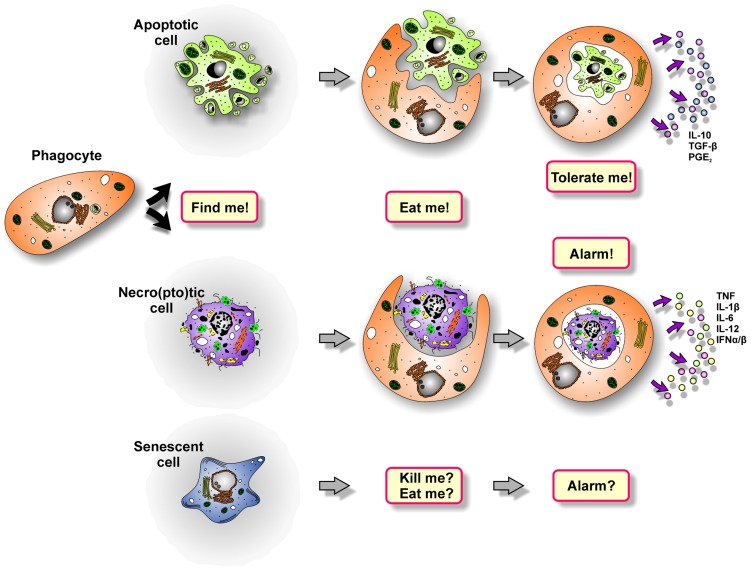
**The multi-step process of dying cell clearance**.

The sophisticated process of dying cell removal involves distinct phases: phagocyte recruitment, prey cell engulfment, and the post-phagocytic response (Ravichandran, 2010; **Figure 2**).

### PHAGOCYTE RECRUITMENT

**FIGURE 3:**
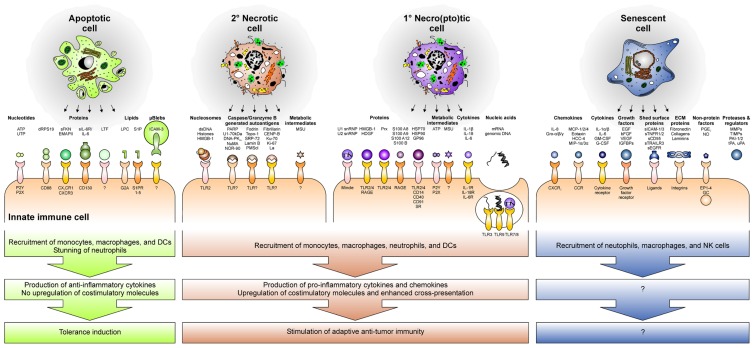
**The secretome and releasate of cells undergoing different forms of cell death and senescence recruit immune cells and skew the immune response toward tolerance induction or anti-tumor immunity**.

Phagocyte recruitment is accomplished by the release of soluble “find-me” signals from the dying cell (Munoz et al., 2010b; Peter et al., 2010; **Figure 3**). In case of apoptotic cells, these “find-me” signals comprise different molecular entities. As such, nucleotides, like ATP and UTP, have been described to trigger monocyte/macrophage recruitment via the purinergic P2Y_2_ receptor (Elliott et al., 2009). Released proteins, including a covalently linked dimer of ribosomal protein S19 (dRPS19), endothelial monocyte activating polypeptide II (EMAP-II), soluble fractalkine (sFKN), and the ectodomain of the IL-6 receptor (sIL-6R) also contribute to phagocyte attraction and involve their cognate receptors CD88, CXCR3, CX_3_CR1, and CD130 (Nishiura et al., 1998; Hou et al., 2006; Chalaris et al., 2007; Truman et al., 2008). Phospholipids, such as lysophosphatidylcholine (LPC) and sphingosine-1-phosphate (S1P), complement the molecular spectrum of apoptotic cell-derived “find-me” signals and have been reported to stimulate monocyte/macrophage chemotaxis via the G-protein coupled receptor G2A or the family of the S1P receptors (S1PR1–5), respectively (Lauber et al., 2003; Gude et al., 2008; Peter et al., 2008). Finally, also micro-blebs carrying ICAM-3 have been assigned a role in phagocyte recruitment by apoptotic cells (Segundo et al., 1999; Torr et al., 2012). Amongst the mediators being liberated during apoptosis, lactoferrin (LTF) plays a special role. Its *de novo* expression and release by apoptotic cells have been described, but in contrast to the “find-me” signals described above, LTF exerts the function of a granulocytic anti-attraction or “keep-out” signal, since it exerts a deterring effect on neutrophils and eosinophils and prevents them from invading the area of apoptotic cell death (Bournazou et al., 2009).

Of note, all these factors are released and/or secreted while the plasma membrane is still intact – a crucial hallmark of bona fide apoptosis, which fundamentally discriminates it from necro(pto)sis. In the course of the latter, the disintegration of the plasma membrane liberates the intracellular contents, and the most abundant factors within this “releasate” act as endogenous danger signals or damage-associated molecular patterns (DAMPs) that “inform” the immune system about the tissue damage (Matzinger, 1998; Lotze et al., 2007). The majority of danger signals have been reported to exert pleiotropic effects on the immune system, with phagocyte recruitment being one of them. As such, high mobility group box 1 protein (HMGB-1) and its close relative hepatoma-derived growth factor (HDGF), peroxiredoxins (Prx), members of the S100 protein family, and heat shock proteins (HSPs) can stimulate monocyte/macrophage attraction by engaging the Toll-like receptors 2 and 4 (TLR2/4), the receptor for advanced glycation end products (RAGE), and members of the scavenger receptor (SR) family (Eue et al., 2000; Bianchi and Manfredi, 2007; Tsan and Gao, 2009; Shichita et al., 2012). Additionally, ATP and uric acid, which readily forms monosodium urate (MSU) crystals in the extracellular space, have been assigned a phagocyte attracting danger signal function. Yet, the receptor for MSU crystals, if there is any, remains to be identified (Shi et al., 2010).

In case of post-apoptotic, secondary necrotic cells, the process of phagocyte recruitment is only poorly understood. One could argue that the repertoire of soluble mediators released by secondary necrotic cells should be a sum of the factors released by apoptotic and primary necro(pto)tic cells. However, it has to be taken into account that dRP S19, EMAP-II, sFKN, sIL-6R, LPC, S1P, and micro-blebs are released early during apoptosis, and according to the ubiquitous presence of degrading enzymes in the extracellular space the stability of these compounds is presumably rather limited. Hence, it is questionable if these mediators are still present and active after the transition into secondary necrosis. Moreover, the factors liberated during primary and secondary necrosis should differ essentially, since the key danger signals described in the context of primary necrosis undergo modifications during apoptosis: ATP is consumed and intracellular proteins are proteolytically processed, which might enhance as well as abolish their danger signal function (Galluzzi et al., 2012). Yet, available data on this issue are limited. From the present point of view, the most likely candidates for secondary necrotic cell-derived “find-me” signals are MSU crystals, since the amount of releasable uric acid – owing to the degradation of chromatin – supposedly is even higher than in the case of primary necrosis, and HMGB-1 associated with secondary necrotic cell-derived nucleosomes (Urbonaviciute et al., 2008; Shi et al., 2010). In addition, annexin A1 has very recently been shown to translocate to the outer leaflet of the plasma membrane and to be proteolytically processed into a peptide, which subsequently is released and stimulates monocytes/macrophage chemotaxis during secondary necrosis (Blume et al., 2009, 2012).

### DYING CELL ENGULFMENT

When phagocytes reach the dying cells, they identify and recognize their prey by “eat-me” signals, which are exposed on the cell surface. For apoptotic cells, the dominant “eat-me” signal appears to be phosphatidylserine (PS), an anionic phospholipid that during apoptosis translocates from the inner to the outer leaflet of the plasma membrane (Fadok et al., 1992; Krahling et al., 1999). PS is recognized directly by specific PS receptors, including brain angiogenesis inhibitor 1 (BAI-1), members of the T cell Ig and mucin family (TIM-1, -3, and -4), and the stabilins 1 and 2 (Kobayashi et al., 2007; Miyanishi et al., 2007; Park et al., 2007, 2008, 2009; Nakayama et al., 2009). Additionally, PS can be recognized indirectly via the mediation of soluble bridging proteins. They link PS to different engulfment receptors of the phagocyte, and originate from the phagocyte, the dying cell, or the interstitial body fluids, respectively. Milk fat globule EGF factor 8 (MFG-E8) and its close relative developmental endothelial locus 1 (Del-1) are examples for phagocyte-derived bridging proteins and ligate PS to α_ν_β_3/5_ integrins (Hanayama et al., 2002). In addition, annexin A1 can be released by the phagocyte for apoptotic cell opsonization, but it has also been reported to be the prototypical apoptotic cell-derived bridging protein, although its corresponding phagocyte receptor still has to be identified (Arur et al., 2003; Fan et al., 2004). The serum proteins β_2_-glycoprotein 1 (β_2_GP1), protein S, and growth arrest-specific gene 6 (Gas6) contribute to PS bridging as well and involve members of the LDL receptor-related protein family (LRP), or the protein tyrosine kinases Mer, Axl, and Tyro3, respectively (Ishimoto et al., 2000; Lu and Lemke, 2001; Scott et al., 2001; Anderson et al., 2003; Wu et al., 2005; Rothlin et al., 2007; Maiti et al., 2008; Xiong et al., 2008). Aside from externalized PS, several other “eat-me” signals on the surface of apoptosing cells have been described: sites resembling oxidized low density lipoprotein particles and sites binding thrombospondin-1, collectins or complement proteins have been reported to engage SRs, α_ν_β_3/5_ integrins, collectin and complement receptors in order to trigger dying cell engulfment. Finally, also the inactivation of “don’t-eat-me” signals, such as CD31 or CD47 and its binding partner SIRPα, which prevent viable cells from mistakenly being ingested, contributes to proper dying cell recognition (Brown et al., 2002; Gardai et al., 2005).

In case of primary and secondary necrosis the mechanisms of dying cell recognition and engulfment are not as well understood as in the case of apoptosis. However, it appears that complement opsonization rather represents a hallmark of secondary necrosis than of early apoptosis, and the same seems to apply to annexin A1 externalization (Gaipl et al., 2001; Blume et al., 2009). Moreover, although mechanistically different from PS translocation during apoptosis, ruptures or holes in the plasma membrane lead to an exposure of PS by primary and secondary necrotic cells. Consequently, PS has also been attributed a role in necrotic cell engulfment (Brouckaert et al., 2004; Bottcher et al., 2006). Of note, very recently the first specific “eat-me” signal of primary necrotic cells has been identified: exposed actin filaments, which are recognized by the C-type lectin Clec9A (Sancho et al., 2009; Ahrens et al., 2012; Zhang et al., 2012).

Ligation of engulfment receptors triggers rearrangements in the actin cytoskeleton and thus the internalization of the dying cell. However, the underlying molecular mechanisms are only poorly understood and may differ fundamentally according to the structure of the respective receptor and its downstream signaling cascades. Probably the most detailed data are available for BAI-1, a seven transmembrane domain G-protein coupled receptor that signals via the evolutionarily conserved ELMO1–Dock180–Rac complex (Park et al., 2007). Undoubtedly, further studies are required in order to dissect the signaling cascades of each engulfment receptor. On the one hand this will help to understand why the morphology of apoptotic and necrotic cell ingestion is so different from each other, with apoptotic cells being phagocytosed by a “zipper”-like mechanism and necrotic cells – together with a substantial amount of extracellular material – being taken up by macropinocytosis (Krysko et al., 2006). On the other hand, it will contribute to elucidate, why the phagocytosis of apoptotic and necrotic cells is so fundamentally different in terms of the immunological outcome.

### THE POST-PHAGOCYTIC IMMUNE RESPONSE

After engulfing dying cells, macrophages and DCs exert discrepant functions regarding the subsequent immune response. Whereas macrophages predominantly degrade the phagocytic cargo and shape the cytokine milieu for other immune cells, DCs traffic the ingested material to MHC-II- or MHC-I-dependent pathways of antigen-presentation or cross-presentation, respectively. Of note, the immunological outcome of dying cell phagocytosis fundamentally differs depending on the type of death the internalized prey has previously undergone (**Figure 2**). It can be said very simplistically that the uptake of apoptotic cells blunts pro-inflammatory and induces anti-inflammatory cytokine production in macrophages, including IL-10, TGF-β, and PGE_2_ (Voll et al., 1997; Fadok et al., 1998). DCs take up apoptotic material and process, present, or cross-present apoptotic cell-derived antigens to T cells, thus resulting in the induction of immune tolerance (Albert et al., 1998; Stuart et al., 2002; McGaha et al., 2011). The “tolerate-me” signals involved in this complex process are currently being elucidated, and PS, which is exposed on the apoptotic cell surface, apparently is of pivotal importance in this regard (Huynh et al., 2002; Chen et al., 2004; Doffek et al., 2011). However, due to holes in the plasma membrane, PS is also exposed by primary and secondary necro(pto)tic cells, but in this case dying cell engulfment triggers a potent pro-inflammatory immune response. So, somehow the tolerogenic effect of PS must be overridden. Exposed F-actin, which ligates Clec9A, efficiently stimulates cross-priming of damaged cell-derived antigens, but alone it is not sufficient to induce an adaptive immune response to dying cells (Ahrens et al., 2012). Evidence for the nature of the required molecules comes from a study reporting on the lack of pro-inflammatory responses against necrotic cells, which have been separated from their secretome and/or releasate (Brouckaert et al., 2004). Hence, it is obviously the plethora of molecules released during primary or secondary necro(pto)sis that tip the scale. Different studies have shown that these liberated components, including HMGB-1-decorated nucleosomes, HSPs, ATP, ribonucleosome particles, and others, trigger TLR-, C-type lectin- and nod-like receptor-signaling, which is essential to promote pro-inflammatory cytokine production in macrophages and efficient cross-priming in DCs (Rovere-Querini et al., 2004; Blander, 2008; Ghiringhelli et al., 2009; Aymeric et al., 2010; Garaude et al., 2012; **Figure 3**). This is further substantiated by research in the field of autoimmunity as defects in the process of apoptotic cell clearance and the subsequent accumulation of secondary necrotic debris have been shown to represent hallmarks in the etiopathogenesis of chronic inflammatory and autoimmune diseases, such as systemic lupus erythematosus (SLE; Herrmann et al., 1998; Baumann et al., 2002; Gaipl et al., 2005, 2007a; Munoz et al., 2010a). Moreover, mice deficient in different engulfment genes or DNase II, which is required for the proper degradation of dying cell-derived DNA, reveal an accumulation of dying cell debris and develop a late-onset autoimmune phenotype, including the typical interferon (IFN) signature, closely resembling human SLE (Botto, 1998; Le et al., 2001; Cohen et al., 2002; Hanayama et al., 2006; Kawane et al., 2006).

**FIGURE 4:**
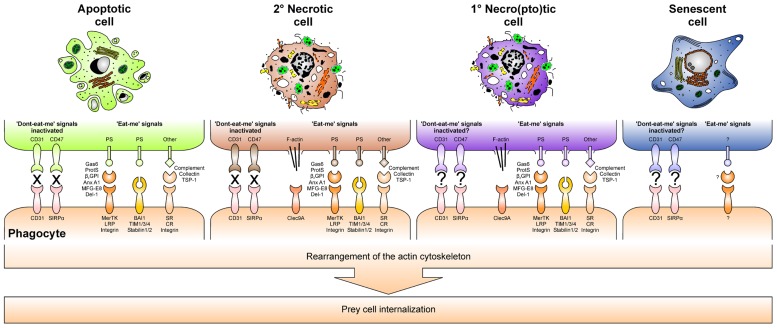
**“Eat-me” signals and inactivated “don’t-eat-me” signals together with their respective phagocyte receptors orchestrate dying cell engulfment**.

In contrast to apoptotic and necro(pto)tic cells, the clearance of senescent cells is only poorly understood. Yet, a pioneering study has shown that senescence induction in established liver carcinomas results in the recruitment of macrophages, neutrophils, and natural killer (NK) cells, and this was sufficient for the clearance of the senescent tumor cells (Xue et al., 2007). However, which factors of the senescence-associated secretome are involved in this scenario (**Figure 3**), if senescent cells are “eaten up alive” or first are actively killed and then phagocytosed, and which “eat-me” signals play a role in this context, remains elusive (**Figure 4**; Kuilman and Peeper, 2009; Coppe et al., 2010). Moreover, senescence induction in pre-cancerous hepatocytes was observed to stimulate an adaptive immune response depending on the interaction of antigen specific CD4^+^ T cells and macrophages (Kang et al., 2011). Whether this might be of use for the induction of anti-tumor immunity in established cancers has to be further evaluated.

It should be noted that the form of cell death or cell stress does not only shape the subsequent immune response, it has also profound impact on the surviving neighboring cells. In this regard, radiotherapy-induced apoptosis has been shown to potently promote tumor cell repopulation via mechanisms involving the caspase-3-dependent cleavage and activation of the calcium-independent phospholipase A_2_ (iPLA_2_) and the subsequent production of prostaglandin E_2_ (PGE_2_; Huang et al., 2011; Lauber et al., 2011). Moreover, DAMPs released from necro(pto)tic cells and particularly HMGB-1 have been reported to stimulate autophagy as a mechanism of programed cell survival and thus to confer resistance to radiotherapy and chemotherapy in different models of leukemia, pancreatic, and colon cancer (Tang et al., 2010a,b; Liu et al., 2011a,b). As mentioned above, also the senescence-associated secretome contains factors, which can suppress or promote the proliferation of surviving neighboring cells, respectively (Kuilman and Peeper, 2009; Coppe et al., 2010). Presumably, these proliferation-stimulating effects of dying cells and their releasates represent integral parts of a conserved wound-healing program, which controls tissue regeneration and repair under physiological conditions. However, in the context of cancer therapy they might be counterproductive as therapy-induced tumor cell repopulation and/or therapy resistance would strongly interfere with the therapeutic aim of tumor eradication.

## DYING CELL CLEARANCE AND THE INDUCTION OF ANTI-TUMOR IMMUNE RESPONSES IN RESPONSE TO RADIOTHERAPY

**FIGURE 5:**
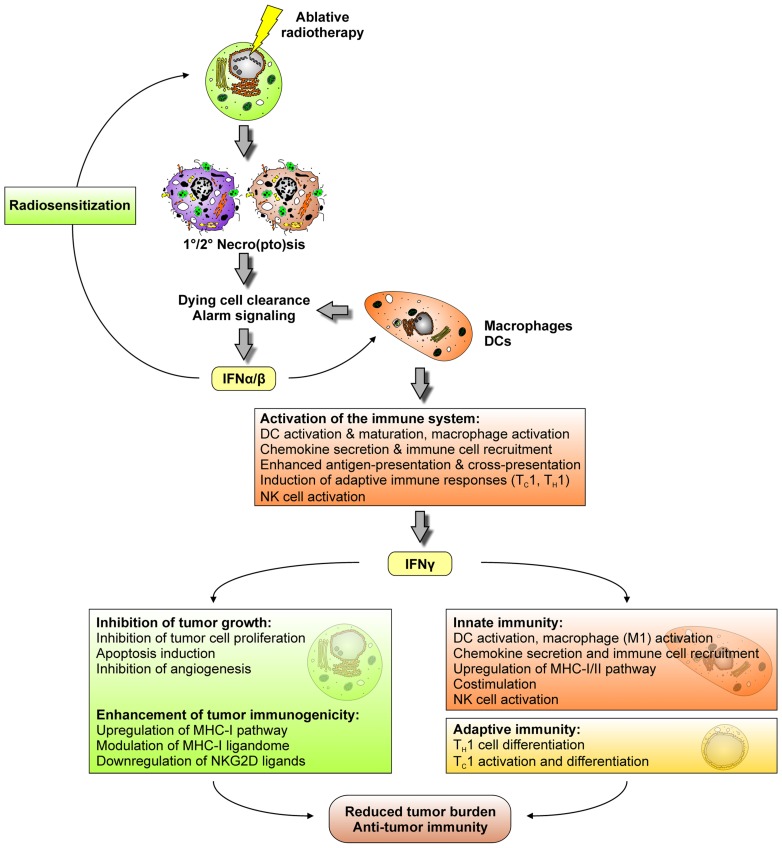
**Ablative radiotherapy stimulates a cascade of interferons, which contributes to a reduction in tumor burden and the induction of anti-tumor immunity**.

It is well acknowledged that the induction of tumor cell death and the abrogation of clonogenic survival by ionizing irradiation are key determinants of its therapeutic success. However, there is accumulating experimental evidence that particularly in the context of ablative radiotherapy, during which radiation is applied in high single doses of 10 Gy or more, complex immune mechanisms contribute to tumor regression. One of the initial reports on this issue showed that local high dose radiation therapy of transplanted mouse B16 melanoma stimulates the generation of tumor antigen-specific, IFN-γ producing T cells (Lugade et al., 2005). In the same mouse model, ablative, but not fractionated radiotherapy was observed to drastically enhance T cell priming in tumor draining lymph nodes paralleled by a reduction/eradication of the primary tumor as well as distant metastases in a CD8^+^ T cell-dependent manner (Lee et al., 2009). A mechanistic explanation of how these T cells are primed was provided by a recent study showing that the intra-tumoral production of IFN-α/β in response to high dose radiotherapy enhances the cross-presenting capacity of tumor infiltrating DCs (Burnette et al., 2011). Hence, it appears that ablative radiotherapy triggers a temporal cascade of IFNs, which is well-known from the field of tumor immunoediting, where IFN-α/β produced by CD11c^+^ cells (presumably DCs and macrophages) enhances the cross-priming activity of CD8α^+^ DCs, thus stimulating the generation of IFN-γ producing CD8^+^ T cells, and finally tumor rejection (**Figure 5**; Diamond et al., 2011; Fuertes et al., 2011). Notably, there is a clear difference in how IFN-α/β and IFN-γ contribute to a reduction in tumor burden. IFN-α/β predominantly affects macrophages, DCs, and NK cells leading to their activation and/or maturation, the upregulation of chemokine expression, the enhancement of antigen-presentation and cross-presentation by DCs and a robustly augmented induction of adaptive immune responses (Dunn et al., 2006). Consequently, in the context of cancer immunoediting, IFN-α/β-responsiveness is specifically required in DCs and macrophages, whereas it appears to be dispensable in tumor cells (Diamond et al., 2011; Fuertes et al., 2011). Nevertheless, tumor rejection in response to radiotherapy might also be improved – at least in part – by a direct effect of IFN-α/β on the tumor via its reported capacity of radiosensitization (Morak et al., 2011). In strong contrast to IFN-α/β, tumor cell responsiveness to IFN-γ obviously is an essential prerequisite for the development of anti-tumor immune responses. Various anti-tumor mechanisms have been reported to be exerted by IFN-γ, including inhibition of tumor cell proliferation, apoptosis induction, inhibition of angiogenesis, and an overall enhancement of tumor immunogenicity as characterized by an upregulation of the MHC-I pathway, modulation/extension of the MHC-I ligandome, and downregulation of NKG2D ligands (Dunn et al., 2006; Reits et al., 2006; Lugade et al., 2008). Notwithstanding these direct effects on the tumor, IFN-γ is key for the stimulation of an anti-tumor immune response. As such, IFN-γ is vitally involved in T_H_1/T_C_1 cell differentiation/activation, and it exerts similar effects as IFN-α/β in terms of innate immune cell activation and the promotion of DC-mediated antigen cross-presentation (Dunn et al., 2006).

Once more it should be emphasized that this IFN-controlled cascade of innate and adaptive immune responses was only observed in case of ablative but not conventional, fractionated radiotherapy (Lee et al., 2009). A feasible explanation for this might be that ablative and fractionated radiotherapy trigger different modalities of tumor cell death with only high dose irradiation stimulating primary and/or secondary necro(pto)sis. The corresponding liberation of danger signals, including HMGB-1 and ATP, in turn stimulates the TLR4-dependent production of IFN-α/β and other pro-inflammatory cytokines, initiating the above described IFN cascade and the DC-mediated instigation of anti-tumor T cell responses (Apetoh et al., 2007).

**FIGURE 6:**
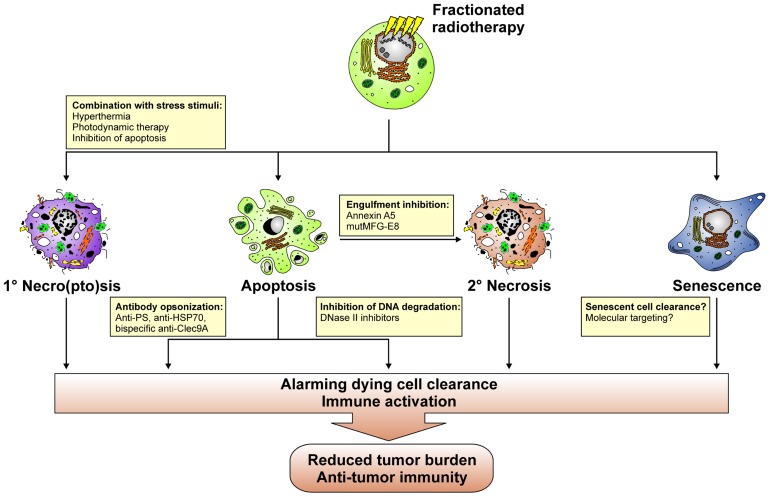
**Putative approaches with which the process of dying cell clearance can be instrumentalized in order to induce anti-tumor immunity in the context of fractionated radiotherapy**.

A key question that arises from an immuno-radiotherapeutic point of view at this point is: How can the process of dying cell clearance be instrumentalized or manipulated in order to enhance the efficacy of fractionated radiotherapy? Various putative approaches can be envisioned in this regard (**Figure 6**). The observations made with high dose ablative radiotherapy suggest that the temporary induction of primary/secondary tumor cell necro(pto)sis might be beneficial. Initial studies on this issue in fact provide evidence that the combination of radiotherapy with hyperthermia results in the induction of an immunogenic type of cell death as characterized by the release of danger signals, including HMGB-1 and HSP70, which foster the maturation of DCs *in vitro* (Schildkopf et al., 2009, 2010, 2011; Mantel et al., 2010). However, it remains to be elucidated, whether this translates into the productive stimulation of anti-tumor T cell responses *in vivo*. Alternatively, radiotherapy might be combined with photodynamic therapy (PDT), since PDT has been reported to induce the expression of HSPs and the release of danger signals (Castano et al., 2006). Alone or in combination with chemotherapy PDT leads to the stimulation of anti-tumor T cell responses *in vivo* (Castano et al., 2008; Kammerer et al., 2011). Yet, if this holds also true for its combination with radiotherapy awaits further clarification. For tumors, which predominantly undergo apoptosis in response to fractionated radiotherapy, caspase inhibition might represent an approach to overcome the tolerogenic nature of this form of cell death, since caspase inhibition in apoptosing cells has been reported to directly trigger necroptosis (He et al., 2009b). However, caspase inhibition might also confer radioresistance due to an overall inhibition of cell death. Therefore, the applicability of this approach has to be very carefully evaluated.

Directly interfering with apoptotic cell clearance in order to promote the accumulation of secondary necrotic tumor cell material might be an alternative approach to instigate the IFN cascade described above and a concomitant anti-tumor immune response. In this regard, annexin A5 and a mutant form of MFG-E8, which no longer is able to bind to the vitronectin receptor, might be valuable tools (Hanayama et al., 2002; Bondanza et al., 2004; Gaipl et al., 2007b; Munoz et al., 2007a,b; Frey et al., 2009). In a mouse model of tumor vaccination, annexin A5 has been shown to impair the uptake of irradiated apoptotic lymphoma cells by macrophages and to specifically target them to CD8α^+^ DCs, thus inducing the release of proinflammatory cytokines and tumor-specific immune memory, which contributed to the regression of growing tumors and conferred resistance against tumor re-challenge (Bondanza et al., 2004). Redirecting dying cell clearance toward Fcγ receptor-mediated phagocytosis also appears a promising strategy for the induction of anti-tumor immune responses. For example, opsonization with autoantibodies has been reported to potently trigger FcyR-dependent phagocytosis of dying cells and subsequent pro-inflammatory cytokine production (Manfredi et al., 1998; Sarmiento et al., 2007; Grossmayer et al., 2008). Hence, “eat-me” signal specific antibodies, including anti-PS antibodies, or tumor antigen specific antibodies, like anti-HSP70 antibodies, might help to utilize pro-inflammatory dying cell phagocytosis for the induction of anti-tumor immunity (Ran et al., 2005; Gehrmann et al., 2008; He et al., 2009a; Stangl et al., 2011a,b). Engineering these antibodies into bi-specific antibodies coupling to Clec9A, is of special interest in this regard, since thereby dying tumor cell material could be specifically targeted toward the recycling endosomal compartment, thus favoring cross-presentation in BDCA3^+^ DCs, the alleged human equivalent to mouse CD8α^+^ DCs (Schreibelt et al., 2012). Seminal data obtained with different mouse models of viral vaccination point to the direction that this represents a successful approach of inducing vaccine-specific immune responses (Idoyaga et al., 2011; Iborra et al., 2012; Zelenay et al., 2012). It should be noted that Clec9A ligation alone is not sufficient to induce DC maturation and a concomitant adaptive immune response against dying cells (Ahrens et al., 2012). Coligation of additional receptors for DAMPs instead appears to be required. Research in the field of autoimmunity has convincingly shown that dying cell-derived nucleosomal material is very potent in this regard (Frisoni et al., 2007; Urbonaviciute et al., 2008). Hence, interfering with DNase II activity, which in phagocytes mediates the proper degradation of engulfed prey cell DNA, could represent a promising strategy to stimulate the described IFN cascade required for the induction of productive immune responses and well-known for its crucial role in the etiopathogenesis of autoimmune diseases, such as SLE (Kawane et al., 2006; Marshak-Rothstein, 2006; Deng and Tsao, 2010). To this end, cell-permeable, small molecule inhibitors of DNase II would be helpful, and it remains to be elucidated whether such compounds can technically be developed. Finally, further studies are required to improve and expand our knowledge on the process of senescent cell clearance with special focus on its applicability and potential utilization for the enhancement of tumor immunogenicity in the context of fractionated radiotherapy.

## CONCLUSION

Undoubtedly, the induction of tumor cell death and the abrogation of clonogenic survival by ionizing irradiation are key to its therapeutic success. However, pioneering immuno-radiotherapeutic studies have convincingly shown that a contribution of complex immune mechanisms – particularly in the context of ablative radiotherapy – can no longer be neglected. The clearance of dying tumor cells by phagocytic cells of the innate immune system represents a crucial initiating step in this scenario. Valuable lessons can be learned from research in the field of autoimmunity on how the form of cell death skews the subsequent immune response, which cell types and molecules are involved in this context, and how these might be utilized for the induction of productive anti-tumor immune responses in combination with radiotherapy. Notably, these immune responses would not only target the local tumor but could also reach distant, out-of-field metastases. To this end, combined, multi-modal treatment regimes have to be developed with the capacity to induce immunogenic forms of tumor cell death and concomitantly activate the immune system. The correct timing will be an essential parameter affecting the therapeutic success of these approaches, since the priming of adaptive immune responses takes place in the tumor draining lymph nodes, which due to the risk of tumor cell spread and metastasis formation are commonly removed by surgery. Hence, it might be worth considering – carefully and on an individualized basis – to postpone lymph node surgery until the initial anti-tumor immune priming has successfully been accomplished. Not only in this regard, the close collaboration of clinical radiation oncologist, surgeons, radiobiologists, molecular oncologists, and immunologists is indispensable in order to develop and optimize the personalized therapeutic regime with the highest benefit for each individual patient.

## Conflict of Interest Statement

The authors declare that the research was conducted in the absence of any commercial or financial relationships that could be construed as a potential conflict of interest.
